# Prevalence and Predictors of Contrast-Induced Nephropathy (CIN) in Patients with ST-Segment Elevation Myocardial Infarction (STEMI) Undergoing Percutaneous Coronary Intervention (PCI): A Meta-Analysis

**DOI:** 10.1155/2019/2750173

**Published:** 2019-08-25

**Authors:** Huan He, Xiao-Rui Chen, Yun-Qing Chen, Tie-Sheng Niu, Yi-Meng Liao

**Affiliations:** ^1^Department of Cardiology, The Second Affiliated Hospital of Chongqing Medical University, Chongqing 400010, China; ^2^Department of Cardiology, Shengjing Hospital of China Medical University, Shenyang 110022, China

## Abstract

**Background:**

Contrast-induced nephropathy (CIN) becomes more and more frequent after percutaneous coronary intervention (PCI) in patients with ST-segment elevation myocardial infarction (STEMI). There have been no reported meta-analyses to determine the role of these risk factors in predicting CIN in patients with STEMI undergoing PCI. So we made this meta-analysis to summarize the incidence of CIN in patients with STEMI undergoing PCI and to study associations between CIN and several risk factors that are mentioned in most prevention guidelines.

**Hypothesis:**

The overall incidence of CIN in patients with STEMI undergoing PCI is not low. Many risk factors could influence the occurrence of CIN, such as hypertension, diabetes mellitus (DM), and lower estimated glomerular filtration rate.

**Methods:**

Databases, including PubMed, Embase, Cochrane Library, China National Knowledge Infrastructure (CNKI), and Chinese BioMedical (CBM), were searched for articles published before May 21, 2019, to identify all relevant studies on CIN. The pooled data were analyzed using either fixed-effects or random-effects models depending on heterogeneity (assessed via the *I*^2^ index).

**Results:**

Twelve articles encompassing a total of 6342 patients were included. The overall pooled CIN incidence was 13.3% (95% CI: 10.4–17.1). The forest plots showed positive associations between CIN and the presence of hypertension, diabetes mellitus, history of prior myocardial infarction, age, damaged left anterior descending artery, Killip class ≥2, decreased left ventricular ejection fraction, lower estimated glomerular filtration rate, and left ventricular ejection fraction <40%; the odds ratios for these factors were 1.85 (95% CI: 1.57–2.18; *p* < 0.00001), 1.83 (95% CI: 1.47–2.29; *p* < 0.00001), 2.14 (95% CI: 1.46–3.14; *p* < 0.0001), 7.79 (95% CI: 5.24–10.34; *p* < 0.00001), 1.92 (95% CI: 1.15–3.22; *p*=0.01), 3.12 (95% CI: 2.21–4.40; *p* < 0.00001), −6.15 (95% CI: −9.52 to −2.79; *p*=0.0003), −15.06 (95% CI: −24.75 to −5.36; *p*=0.002), and 5.53 (95% CI: 1.10–27.95; *p*=0.04), respectively.

**Conclusion:**

The overall incidence of CIN in patients with STEMI undergoing PCI was not low and was closely associated with hypertension, diabetes mellitus, history of prior myocardial infarction, age, damaged left anterior descending artery, Killip class ≥2, decreased left ventricular ejection fraction, lower estimated glomerular filtration rate, and left ventricular ejection fraction <40%.

## 1. Introduction

Percutaneous coronary intervention (PCI) has become increasingly important and common in the treatment of ST-segment elevation myocardial infarction (STEMI), resulting in fewer ischemic complications, more survival myocytes, preserved ventricular function, and improved survival of patients when compared with treatment via pharmacologic reperfusion with fibrinolytic agents [1, 2]. The incidence rates of major adverse cardiovascular events (MACEs) are higher in patients with STEMI who undergo PCI compared with patients with other types of coronary heart disease, such as non-ST-segment elevation acute coronary syndrome and stable angina [3].

As a result, contrast-induced nephropathy (CIN), a relatively infrequent complication after PCI in patients with STEMI, has attracted increasing attention [4]. CIN may lead to worse clinical outcomes, including prolonged hospitalization, increased costs, repeat revascularization, and short- and long-term mortality. The mechanisms of CIN are vasoconstriction, oxidative stress, medullary ischemia, and direct toxic effects of contrast media (CM) [5, 6]. There are no effective ways to prevent CIN although some reports indicate that hydration to patients with STEMI can reduce CIN. Therefore, it is particularly important to recognize risk factors as early as possible while perioperation to prevent the incidence of CIN [7, 8]. Shira I. Moos concluded from his meta-analysis that the mean incidence of contrast-induced nephropathy (CIN) after intravenous iodinated CECT was low and was associated with renal insufficiency, diabetes, the presence of malignancy, old age, and NSAIDs use [9]. What about the incidence of CIN and related risk factors in patients with ST-segment elevation myocardial infarction (STEMI) undergoing percutaneous coronary intervention (PCI)? Samue Goussot conducted a study and found that factors such as hypertension and diabetes mellitus were not associated with an increased risk for CIN, while Stylianos A. Pyxaras found that hypertension and diabetes mellitus could be independent predictors for CIN [8, 10]. Therefore, the role of these risk factors in predicting CIN in patients with STEMI undergoing PCI remains controversial. To the best of our knowledge, there have been no reported meta-analyses to determine the role of these risk factors in predicting CIN. Thus, these meta-analyses were conducted to elucidate the role of these risk factors and to summarize the incidence of CIN in patients with STEMI undergoing PCI.

## 2. Materials and Methods

### 2.1. Search Strategy and Study Selection

We searched many databases, including PubMed, Embase, Cochrane Library, China National Knowledge Infrastructure (CNKI), and Chinese BioMedical (CBM), for articles published before May 21, 2019, to identify all relevant studies on CIN. We searched studies using the following key words: contrast-induced nephropathy (Title, Abstract, and Keyword), contrast induced acute kidney injury (Title, Abstract, and Keyword), cardiac catherization (Title, Abstract, and Keyword), and percutaneous coronary intervention (Title, Abstract, and Keyword). The detailed searching strategy is shown in the Appendix.

### 2.2. Inclusion and Exclusion Criteria

The inclusion criteria were as follows: (1) written in English or Chinese, (2) patients with STEMI underwent PCI, (3) a prospective follow-up study, and (4) CIN incidence, and risk factors were clearly presented.

The exclusion criteria were as follows: (1) duplicate publication (most recent paper was included for analysis), (2) ICU patients included, and their data could not be separately identified; (3) animal experiment, case report, meeting report, review, and studies on prevention [11, 12].

Two reviewers (Huan He and Xiao-Rui Chen) independently searched databases, included and excluded papers, and assessed the data on methodological assessment. A third reviewer (Yun-Qing Chen) was consulted if the two reviewers could not reach the same conclusion.

### 2.3. Methodological Assessment

We used the Delphi list for randomized controlled trials (RCTs) and the QUADAS-2 tool to finish the methodological assessment of the included studies [9, 13]. The following characteristics were assessed: (1) whether the study was a cohort or RCT, (2) whether the study was a single centre or multicenter study, (3) whether a consecutive or random sample of patients was enrolled, (4) whether inclusion/exclusion criteria were specified, (5) whether the spectrum of patients was representative in real-life practice, (6) whether the detail of the contrast medium was described, (7) whether there was enough time between contrast medium administration and follow-up (performed within 2–4 days, 48–92 h), and (8) whether there were relevant risk factors in the study [14, 15].

### 2.4. Statistical Analysis

To calculate the CIN incidence, we collected data on the number of patients with and without CIN in the selected studies. The incidence was presented as a percentage per study with a corresponding 95% confidence interval (CI). When calculating the incidence of contrast nephropathy, we considered the different definitions of contrast nephropathy used in different studies. We used the *I*^2^ index to test the heterogeneity of the incidence. A random-effects model was used to pool the CIN incidence when *I*^2^ ≥ 25%, while a fixed-effects model was used when *I*^2^ < 25%. We carried out this analysis on logit-transformed incidence because it is assumed to follow a normal distribution in each study, and therefore, we calculated the mean logit CIN incidence with corresponding standard errors. After antilogit transformation, we obtained pooled estimate of CIN incidence (95% CI). All of the abovementioned analyses were executed by using Stata/SE 12.0 software [9, 16, 17].

To elucidate the association between CIN and risk factors, odds ratios (ORs) were calculated based on the 2 × 2 tables. We used the fixed-effects model if *I*^2^ was less than 25%; we used the random-effects model if *I*^2^ higher than 25%. The results are presented in forest plots. Statistical significance for the association was set at *p* = 0.05. We analyzed these data using Cochrane RevMan software (version 5.0) [18, 19].

## 3. Results

### 3.1. Search Strategy and Study Selection

**Figure 1:**
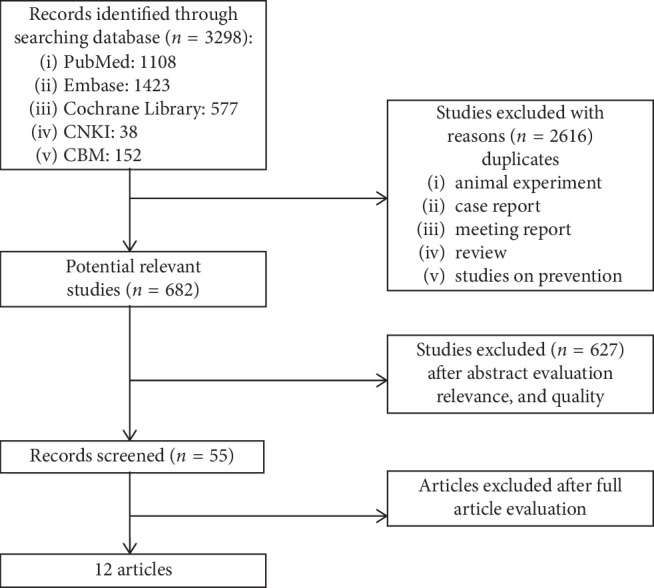
The results of the search strategy, study selection, and inclusion.

We obtained 3298 papers after searching in the databases, in which only 682 papers were left after excluding duplicates, case reports, comments, letters, reviews, conference papers, and studies on prevention. There were 55 papers left after reading the abstract, and full texts were retrieved for further selection. Only 12 papers were found at last because the other 43 papers were not prospective follow-up studies (Figure1).

### 3.2. Methodological Assessment

All 12 papers were cohort studies, in which two papers excluded all patients with renal insufficiency defined as estimated glomerular filtration rate [eGFR] <60 mL/min/1.73 m^2^ or creatinine clearance <60 mL/min. All papers presented their relevant data with 95% CIs. We present the results of the methodological criteria in detail in Table 1 [12].

### 3.3. Overall CIN Incidence

Three papers defined CIN as an increase in serum creatinine ≥25% from the baseline value within the 72-hour period; four papers defined CIN as an increase in serum creatinine ≥0.5 mg/dL; three papers defined CIN as an increase in serum creatinine of ≥25% or ≥0.5 mg/dL; one paper defined CIN as an increase in serum creatinine >25% or a decrease in the estimated glomerular filtration rate (eGFR) <25%; and one paper defined CIN as a rise in serum creatinine >26.5 *μ*mol/L or >50%. CIN was defined within 48–72 h after PCI in twelve papers.

The overall pooled incidence of CIN was 13.3% (95% CI: 10.4–17.1). We did not present the subgroup analysis of the incidence of CIN in different contrast mediums because there were only 12 papers.

### 3.4. CIN Incidence and Associated Risk Factors

In analyzing the risk factors of CIN, not all twelve papers provided complete data. To acquire the original data, we tried our best to communicate with the authors but did not receive any responses. Therefore, we performed data analyses if 2 × 2 data could be extracted from the studies.

#### 3.4.1. Hypertension

**Figure 2:**
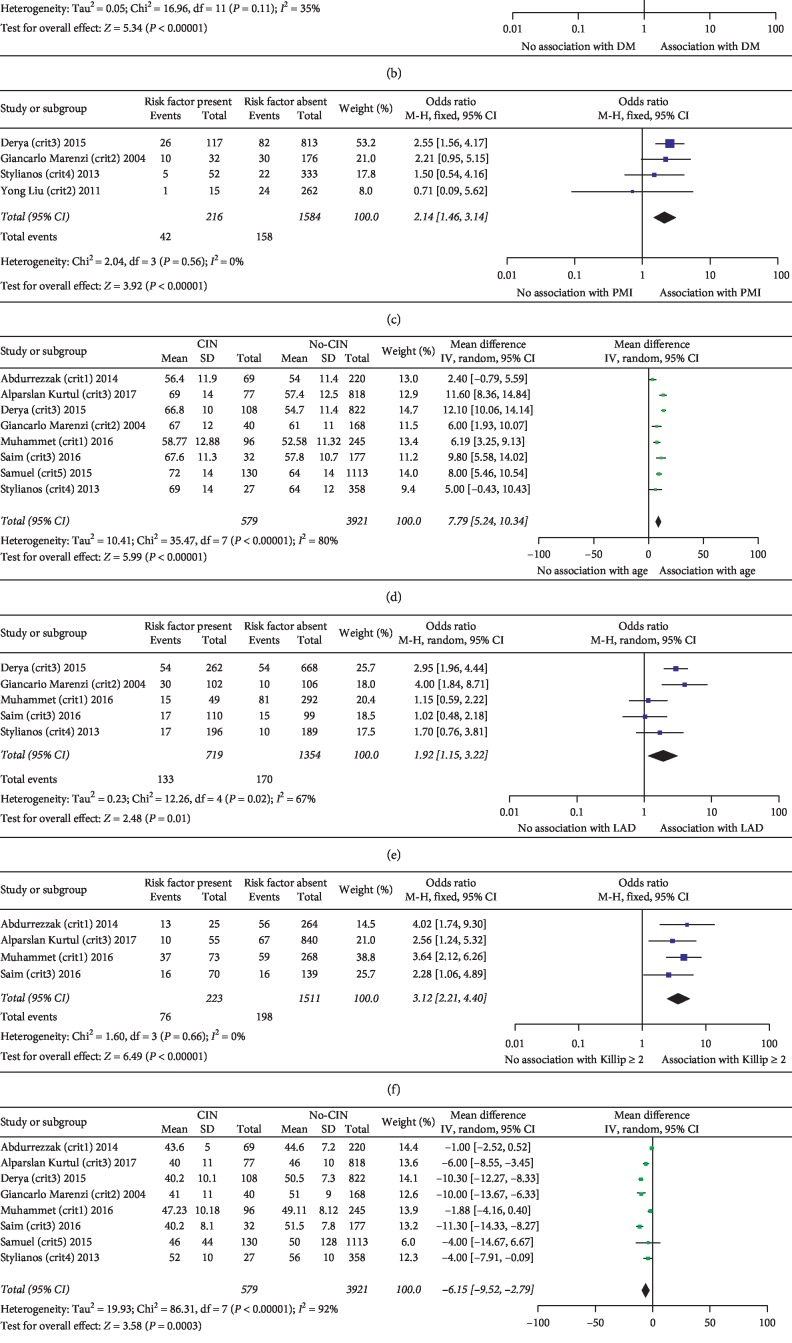
(a)–(i) The positive associations between some risk factors and CIN. HTN, hypertension; DM, diabetes mellitus; PMI, prior myocardial infarction; LAD, left anterior descending; LVEF, left ventricular ejection fraction (%). Crit 1: CIN defined as increase in serum creatinine ≥25%. Crit 2: CIN defined as increase in serum creatinine ≥0.5 mg/dL. Crit 3: CIN defined as increase in serum creatinine of ≥25% or ≥0.5 mg/dL. Crit 4: CIN defined as an increase in serum creatinine >25% or a decrease in the estimated glomerular filtration rate (eGFR) <25%. Crit 5: CIN defined as a rise in serum creatinine >26.5 *μ*mol/L or >50%.

When analyzing the association between hypertension and CIN, we obtained a positive outcome. The OR was 1.85 (95% CI: 1.57–2.18; *p* < 0.00001), and the *I*^2^ value was 0%, which indicated a homogeneous dataset. The forest plot is summarized in Figure 2(a).

#### 3.4.2. Diabetes Mellitus (DM)

In datasets evaluating the association between diabetes mellitus and CIN, an OR of 1.83 (95% CI: 1.47–2.29; *p* < 0.00001) was obtained, with an *I*^2^ value of 35%. These data are shown in Figure 2(b).

#### 3.4.3. The History of Prior Myocardial Infarction

We found that the incidence of CIN was higher in people who had a history of prior myocardial infarction; the OR was 2.14 (95% CI: 1.46–3.14; *p* < 0.0001), and the *I*^2^ value was 0%. These data are shown in Figure 2(c).

#### 3.4.4. Age

The pooled analysis of the relationship between age and CIN showed a nonhomogeneous dataset (*I*^2^ = 80%) and a positive association, OR 7.79 (95% CI: 5.24–10.34; *p* < 0.00001). These data are shown in Figure 2(d).

#### 3.4.5. Damaged Left Anterior Descending Artery

If the target vessels included left anterior descending in patients, then CIN was more likely to occur. In the meta-analysis of the association between damaged left anterior descending and CIN, the OR was 1.92 (95% CI: 1.15–3.22; *p*=0.01), and the *I*^2^ value was 67%. These data are shown in Figure 2(e).

#### 3.4.6. Killip Class ≥2

We found that the OR was 3.12 (95% CI: 2.21–4.40; *p* < 0.00001), and the *I*^2^ value was 0% when evaluating the association between Killip class ≥2 and CIN. The forest plot is shown in Figure 2(f).

#### 3.4.7. Decreased Left Ventricular Ejection Fraction (%)

We found that the left ventricular injection fraction (%) was lower in the CIN group, with an OR of −6.15 (95% CI: −9.52 to −2.79; *p*=0.0003), as shown in the forest plot. The *I*^2^ value was 92%. These data are shown in Figure 2(g).

#### 3.4.8. Lower Estimated Glomerular Filtration Rate (eGFR)

A lower estimated glomerular filtration rate led to an increased likelihood of developing CIN. The OR was −15.06 (95% CI: −24.75 to −5.36; *p*=0.002), and the *I*^2^ value was 49%. These data are shown in Figure 2(h).

#### 3.4.9. Left Ventricular Ejection Fraction (%) <40%

Similar to those with a decreased left ventricular ejection fraction, those with a left ventricular ejection fraction less than 40% were more likely to develop CIN, with an OR of 5.53 (95% CI: 1.10–27.95; *p*=0.04), as shown in the forest plot. The *I*^2^ value was 95%. These data are shown in Figure 2(i).

#### 3.4.10. Other Risk Factors

We found that anemia, triglyceride, total cholesterol, high-density lipoprotein cholesterol (HDL-C), low-density lipoprotein cholesterol (LDL-C), and hemoglobin could not predict the incidence of CIN; the ORs were 2.21 (95% CI: 0.85–5.74; *p*=0.10), −5.64 (95% CI: −19.53–8.25; *p*=0.43), 4.30 (95% CI: −3.75–12.34; *p*=0.30), 1.55 (95% CI: −0.46–3.57; *p*=0.13), 5.99 (95% CI: −0.19–12.17; *p*=0.06), and −1.85 (95% CI: −12.11–8.41; *p*=0.72), respectively. The *I*^2^ values were 71%, 0%, 0%, 15%, 0%, and 95%, respectively. Gender (male), smoking, family history of coronary atherosclerotic heart disease, damaged right coronary artery (RCA), and damaged left circumflex artery (LCA) were not significantly associated with the incidence of CIN. All the data are shown in Table 2.

## 4. Discussion

Our study showed that the incidence of CIN in patients with ST-segment elevation myocardial infarction undergoing PCI was 13.3% (95% CI: 10.4–17.1).

By analyzing many risk factors that we extracted from previous studies, we only found a few risk factors associated with CIN: hypertension, diabetes mellitus, a history of prior myocardial infarction, age, damaged left anterior descending artery, Killip class ≥2, decreased left ventricular ejection fraction, lower estimated glomerular filtration rate, and left ventricular ejection fraction <40%.

Other risk factors such as gender (male), smoking, anemia, family history of coronary atherosclerotic heart disease, triglyceride, total cholesterol, high-density lipoprotein cholesterol (HDL-C), low-density lipoprotein cholesterol (LDL-C), hemoglobin, damaged RCA, and damaged LCA do not appear to have a significant association with the incidence of CIN.

CM (contrast medium) plays a predominant role in CIN, yet specific underlying mechanisms have not been fully elucidated [20, 21]. A decrease of the glomerular filtration rate caused by various mechanisms leads to the occurrence of CIN. CM can damage the renal and vascular endothelium in either direct or indirect ways, including rheological alterations, activation of tubuloglomerular feedback, hypoxia, cytotoxic effects, reactive oxygen species, adenosine, and endothelin mediators [22]. In fact, in addition to renal impairment, older patients are more likely to experience vascular stiffness and impaired endothelial function. Hypertension can lead to ischemia of kidney tissue, loss of nephrons, a reduction of the number of effective nephrons, and a decreased glomerular filtration rate. DM and the administration of CM are both associated with marked alterations of renal physiology, which include changes of the glomerular filtration rate (GFR) and renal hemodynamics, enhanced tubular transport activity and oxygen expenditure, and intensification of medullary hypoxia and reactive oxygen species (ROS) generation [23–25]. Deterioration of cardiac function contributes to hemodynamic instability, which reduces the effective blood flow to the kidney, consequently trigging renin-angiotensin stagnation, activating the sympathetic nervous system and increasing the level of inflammatory factors and oxygen free radicals, all of which contribute to the occurrence and development of CIN [26]. These mechanisms may partly explain why these risk factors including Killip class ≥2 and decreased left ventricular ejection fraction (%) (LVEF) may predict the occurrence of CIN. A history of prior myocardial infarction and a damaged left anterior descending artery may also predict the occurrence of CIN, which may be related to further reductions in LVEF.

Similar to other studies, our meta-analysis also has some limitations. First, there is no uniform definition of CIN, which may influence the incidence of CIN across studies. We did not perform a subgroup analysis of the incidence of CIN because of the low number of studies available for analysis. Second, we also did not take CM into account when analyzing the risk factors due to an insufficient amount of data. Third, compared with elective PCI, primary PCI is associated with a higher incidence of CIN, a complication that is associated with increased in-hospital and long-term morbidity and mortality [27]. We also did not perform a subgroup analysis between elective PCI and primary PCI because all of the patients in all 12 studies underwent primary PCI. Fourth, although some studies have reported that preventive strategies such as hydration or oral statins can reduce the incidence of CIN, we did not analyze the relationship between prevention measures and the occurrence of CIN [28, 29]. Fifth, the pooling of heterogeneous studies with regard to the selection of patients is another well-known limitation. For example, the definition of STEMI, inclusion criteria, and exclusion criteria vary across articles. Finally, not every included article examined all risk factors, and not every article conducted multiple regression analysis. We contacted the corresponding authors of the papers to try to obtain the data we wanted but unfortunately did not succeed. We therefore completed univariate analyses to study associations between risk factors and CIN. Ideally, we would like to perform a multivariate regression analysis which used all risk factors as the independent variables and CIN incidence as the dependent variable. However, the real relationship between CIN and the risk factors in this population could not be demonstrated in this analysis because of the lack of data for risk factors. We cannot assume that, in cases of missing data, risk factors are not present [9].

## 5. Conclusion

The overall incidence of CIN in patients with STEMI undergoing PCI does not seem low. The main risk factors that are likely associated with CIN in patients with STEMI undergoing PCI are hypertension, diabetes mellitus, history of prior myocardial infarction, age, damaged left anterior descending artery, Killip class ≥2, decreased left ventricular ejection fraction, lower estimated glomerular filtration rate, and left ventricular ejection fraction >40%. A large sample size randomized controlled study is needed to support our conclusion.

## Figures and Tables

**Table 1:** Methodological and design criteria of all 12 included articles [9].

Author, year of publication	Type of study^a^	Study design^b^	Patient selection^c^	Inclusion/exclusion criteria^d^	Spectrum of patients^e^	Description contrast administration^f^	Time interval 2–4 days (48–96 h)^g^	Complete veriﬁcation^h^
Abdurrezzak (2014)	Cohort	Single centre	Yes	Yes	No	Yes	Yes	Yes
Alparslan Kurtul (2017)	Cohort	Single centre	Yes	Yes	Yes	Yes	Yes	Yes
Derya (2015)	Cohort	Single centre	*Unclear*	Yes	Yes	Yes	Yes	Yes
Giancarlo (2010)	Cohort	Single centre	Yes	Yes	Yes	Yes	Yes	Yes
Giancarlo Marenzi (2004)	Cohort	Single centre	Yes	Yes	Yes	Yes	Yes	Yes
Muhammet (2016)	Cohort	Single centre	Yes	Yes	No	Yes	Yes	Yes
Nyman U (2008)	Cohort	Single centre	Yes	Yes	Yes	Yes	Yes	Yes
Saim (2016)	Cohort	Single centre	Yes	Yes	Yes	Yes	Yes	Yes
Samuel (2015)	Cohort	Multicenter	Yes	Yes	Yes	Yes	Yes	Yes
Stylianos (2013)	Cohort	Multicenter	Yes	Yes	Yes	No	Yes	Yes
Yong Liu (2011)	Cohort	Single centre	Yes	Yes	Yes	Yes	Yes	Yes
Yuan-Hui Liu (2017)	Cohort	Single centre	Yes	Yes	Yes	Yes	Yes	Yes

^a^The study was a cohort or randomized controlled trial (RCT). ^b^The study was a single centre or multicentre study. ^c^A consecutive or random sample of patients was enrolled. ^d^Inclusion/exclusion criteria were specified. ^e^The spectrum of patients was representative of the patients who will receive the test in daily practice. ^f^The administration of the contrast medium was described with sufficient details. ^g^The time period between contrast medium administration and follow-up was reasonable (performed within 2–4 days). ^h^The whole (or random) sample underwent follow-up for occurrence/determination of CIN.

**Table 2:** Related data on other risk factors.

Risk factor	Heterogeneity (%)	Risk factor present		Risk factor absent		Odds ratio
		CIN	No. of patients	CIN	No. of patients	
Gender (male) (*n* = 10)	*I* ^2^ = 55	459	3835	178	1336	0.87 [0.63, 1.19] test for the overall effect: *Z* = 0.90 (*p*=0.37)
Smoking (*n* = 8)	*I* ^2^ = 90	233	2036	346	2464	0.66 [0.36, 1.22] test for the overall effect: *Z* = 1.32 (*p*=0.19)
Family history (*n* = 4)	*I* ^2^ = 0	66	617	229	2151	1.01 [0.73, 1.40] test for the overall effect: *Z* = 0.08 (*p*=0.94)
Damaged right coronary artery (RCA) (*n* = 5)	*I* ^2^ = 0	46	565	257	1508	0.49 [0.34, 0.69] test for the overall effect: *Z* = 4.07 (*p* < 0.0001)
Damaged left circumflex artery (LCA) (*n* = 5)	*I* ^2^ = 15	73	623	230	1450	0.70 [0.52, 0.93] test for the overall effect: *Z* = 2.44 (*p*=0.01)

## Appendix

### Search Strategy in Details

PubMed:

#1: percutaneous coronary intervention“[Mesh] 49546.

#2: (((((((((((((((((((((((((((coronary intervention, percutaneous [Title/Abstract]) OR coronary interventions, percutaneous [Title/Abstract]) OR intervention, percutaneous coronary [Title/Abstract]) OR interventions, percutaneous coronary [Title/Abstract]) OR percutaneous coronary interventions [Title/Abstract]) OR percutaneous coronary revascularization [Title/Abstract]) OR coronary revascularization, percutaneous [Title/Abstract]) OR coronary revascularizations, percutaneous [Title/Abstract]) OR percutaneous coronary revascularizations [Title/Abstract]) OR revascularization, percutaneous coronary [Title/Abstract]) OR revascularizations, percutaneous coronary [Title/Abstract]) OR coronary arteriography [Title/Abstract]) OR angiography, coronary [Title/Abstract]) OR angiographies, coronary [Title/Abstract]) OR coronary angiographies [Title/Abstract]) OR cardiac catherization [Title/Abstract]) OR catheter, cardiac [Title/Abstract]) OR catheters, cardiac [Title/Abstract]) OR intracardiac catheters) OR catheter, intracardiac) OR catheters, intracardiac) OR intracardiac catheter) OR cardiac catheter) OR heart catheters) OR catheter, heart) OR catheters, heart) OR heart catheter) OR cardiac catheters 160160.

#3: #1 OR #2 177560.

#4: (((contrast-induced nephropathy [Title/Abstract]) OR contrast induced nephropathy [Title/Abstract]) OR (cin [Title/Abstract] AND nephropathy [Title/Abstract])) OR contrast induced acute kidney injury [Title/Abstract] 2478.

#5: #3 AND #4 1108.
